# Risk Factors for In-Hospital Mortality in Infective Endocarditis

**DOI:** 10.36660/abc.20180194

**Published:** 2020-01

**Authors:** Ana Marques, Inês Cruz, Daniel Caldeira, Sofia Alegria, Ana Catarina Gomes, Ana Luísa Broa, Isabel João, Hélder Pereira

**Affiliations:** 1 Hospital Garcia de Orta EPE Almada - Portugal; 2 Laboratório de Farmacologia Clínica e Terapêutica, Faculdade de Medicina, Universidade de Lisboa Lisbon, Portugal; 3 Instituto de Medicina Molecular, Faculdade de Medicina, Universidade de Lisboa Lisbon, Portugal; 4 Centro Cardiovascular da Universidade de Lisboa - CCUL, Faculdade de Medicina, Universidade de Lisboa Lisbon, Portugal

**Keywords:** Endocarditis, Bacterial/mortality, Hospitalization, Comorbidity, Shock Septic, Heart Failure, Risk Factors, Echocardiography/methods, Cardiac Surgery

## Abstract

**Background:**

Infective endocarditis (IE) is associated with severe complications and high mortality. The assessment of mortality rates and predictors for fatal events is important to identify modifiable factors related to the pattern of treatment, in order to improve outcomes.

**Objectives:**

We sought to evaluate clinical outcomes of patients with IE and to determine predictors of in-hospital mortality.

**Methods:**

Retrospective single-center study including patients with IE admitted during a 10-year period (2006-2015). Data on comorbidities, clinical presentation, microbiology and clinical outcomes during hospitalization were evaluated. Risk factors of in-hospital death were analyzed. A p-value < 0.05 was considered significant.

**Results:**

A total of 134 cases were included (73% males, mean age of 61 ± 16 years-old). Half of them had previous valvular heart disease. Healthcare-associated IE and negative blood-cultures occurred in 22% and prosthetic IE in 25%. The aortic valve was the one most often affected by infection. Staphylococcus aureus was the most commonly isolated microorganism. Forty-four (32.8%) patients underwent cardiac surgery. The in-hospital mortality rate was 31.3% (42 patients). The identified risk factors for in-hospital mortality were Staphylococcus aureus etiology (OR 6.47; 95% CI: 1.07-39.01; p = 0.042), negative blood-cultures (OR 9.14; 95% CI: 1.42-58.77; p = 0.02), evidence of valve obstruction in echocardiography (OR 8.57; 95% CI: 1.11-66.25; p = 0.039), clinical evolution with heart failure (OR 4.98; 95%CI: 1.31-18.92; p = 0.018) or septic shock (OR 20.26; 95% CI: 4.04-101.74; p < 0.001). Cardiac surgery was a protective factor of mortality (OR 0.14; 95% CI 0.03-0.65; p = 0.012).

**Conclusion:**

The risk factors for in-hospital mortality were clinical (heart failure, septic shock), evidence of valve obstruction in echocardiography, Staphylococcus aureus etiology or negative blood cultures. Invasive treatment by surgery significantly decreased the mortality risk.

## Introduction

Infective endocarditis (IE) is associated with severe complications and high mortality, despite the improvements in its medical and surgical management.^1,2^

The diverse nature and evolving epidemiological profile of IE ensure that it remains a diagnostic challenge.^2^ The presentation and evolution of IE is highly variable, depending on host factors (such as existence of previous cardiac disease, prosthetic valves or implanted cardiac device, as well as factors that modulate the immune response), the microorganism involved and the adequacy of the provided treatment (antibiotics, heart failure medical treatment, surgery).^2^

The interplay of these factors results in an in-hospital mortality rate of patients with IE ranging from 15% to 30%.^3-9^

The assessment of mortality rates and predictors for fatal events is important to identify modifiable factors and the pattern of treatment in order to further improve the outcomes. This approach identifies the patients at highest risk of death for whom the level of care should be stepped-up.

Therefore, we aimed to evaluate the clinical outcomes of patients with IE and to determine predictors of in-hospital mortality.

## Methods

A retrospective single-center study was performed, including all consecutive adult patients during a 10-year period (January.2006 to December.2015), in a Portuguese public tertiary general hospital, without on-site cardiac surgery department. The protocol was approved by the institutional review board and local ethics committee.

The population of interest was all cases of definite or possible IE according to the modified Duke criteria,^10^ including those corresponding to patients that had more than one IE episode. For diagnosis purposes, cultural criteria consider as positive cultures during an extended incubation time for blood cultures of up to 21 days, according to the local protocol for IE suspicion. The cases were all identified by hospital discharge codes. The patients were followed until discharge or death (including hospitalization at the surgical center).

Demographic and clinical characteristics, type of endocarditis (native valve, prosthetic valve or device-associated), echocardiographic and microbiological findings, as well as surgical procedure and hospitalization outcomes were retrieved. The sample was characterized using basic descriptive statistic measures.

Patients that died during hospital stay were compared with those that survived regarding their demographic and clinical features, microbiological and echocardiographic findings and hospitalization outcomes.

The primary outcome was all-cause in-hospital mortality. The other adverse outcomes of interest were heart failure (defined as the presence of typical symptoms and signs caused by a structural and/or functional cardiac abnormality, resulting in a reduced cardiac output and/or elevated intracardiac pressures), septic shock (characterized by the presence of Systemic Inflammatory Response Syndrome to an infectious process, with sepsis-induced organ dysfunction or tissue hypoperfusion and persistently arterial hypotension, despite the administration of intravenous fluids), evidence of locally uncontrolled infection or periannular complication (valve destruction or perforation, increasing vegetation size, abscess formation, pseudoaneurysm, valve aneurysm and intracardiac fistula) and embolic events (ischemic stroke, hemorrhagic stroke, mycotic aneurism, myelitis/meningitis, peripheral ischemia and splenic, pulmonary or hepatic infarction or abscesses, diagnosed through computed tomography and/or magnetic resonance imaging, performed according to the clinical suspicion of embolism).

Healthcare-associated IE was defined as IE manifesting more than 48 hours after hospital admission or IE acquired in association with an invasive procedure performed in the 6 months before diagnosis during hospital stay and/or manipulation in a hospital setting.

Valve regurgitation detected at echocardiography included both significant valve regurgitation in native valve IE cases and significant intra and paraprosthetic leaks in prosthetic IE cases.

### Statistical analysis

Categorical variables were presented as frequencies and percentages and were compared using the chi-square test. Continuous variables were expressed as means and standard deviations (SD) and were compared using the independent-samples t-test, after normal distribution was checked using the Kolmogorov-Smirnov test or skewness and kurtosis. Continuous variables with skewed distributions were presented as medians and interquartile ranges (IQR) and a non-parametric method (Mann Whitney U test) was employed.

In order to identify predictors of in-hospital mortality, variables with a p value < 0.1 in the univariate analysis were included in a logistic regression using an enter stepwise method. Two models were performed; one model included all Streptococcal Species and the other included the microorganism *Streptococcus gallolyticus*, since they are variables that are not independent of each other and both had a p value < 0.1 in the univariate analysis. The model predictive performance was tested by assessing its discrimination and its calibration. Discrimination was measured with the area under receiver operating characteristic curve (AUROC) and calibration was measured by using pseudo-R^2^ (Nagelkerke R^2^). The final model defined was that with the highest predictive performance according to the AUROC and pseudo-R^2^.

All reported p values were two-tailed, with a p value < 0.05 indicating statistical significance. The statistical analyses were performed using IBM SPSS Statistics software, version 22.

## Results

### Population characteristics

Between January 2006 and December 2015, 134 cases of infective endocarditis were hospitalized in our center: 101 cases had definite IE and the remaining corresponded to possible IE cases, according to the modified Duke criteria. About 73% of these patients were males, the mean age was 61 ± 16 years. The main clinical characteristics, namely the comorbidities, clinical presentation, microbiology and clinical outcomes of IE cases are summarized in Table 1.

**Table 1:** Population characteristics of infective endocarditis cases (n = 134) and p value of univariate analysis of predictors of in-hospital mortality

Variable	IE Survivors (n = 92)	IE Non-Survivors (n = 42)	Total IE cases (n = 134)	p value
Male gender - nº. (%)	70 (76.1%)	28 (66.7)	98 (73.1%)	0.254
**Age - yrs.**				
mean ±SD	60 ± 17	64 ± 14	61 ± 16	0.177
Min - max	22 - 89	31 - 88	22 - 89	
> 75 yrs. - no. (%)	25 (27.2)	9 (21.4)	34 (25.4)	0.478
**Comorbidities - nº. (%)**				
Arterial Hypertension	47 (51.1)	21 (50)	68 (50.7)	0.907
Valvular heart disease	42 (45.7)	24 (57.1)	66 (49.3)	0.217
Heart Failure	18 (19.6)	16 (38.1)	38 (25.4)	0.022*
Hepatic Disease	24 (26.1)	8 (19)	35 (23.9)	0.413
Diabetes	14 (15.2)	8 (19)	22 (15.8)	0.579
Pulmonary Disease	15 (16.3)	6 (14.3)	21 (15.7)	0.766
Coronary Artery Disease	12 (13)	7 (16.7)	19 (14.2)	0.577
Intravenous drug users	13 (14.1)	5 (11.9)	18 (13.4)	0.763
HIV	12 (13)	6 (14.3)	18 (13.4)	0.804
Chronic Renal Disease	9 (9.8)	7 (16.7)	16 (11.9)	0.254
Intracardiac device	7 (7.6)	3(7.1)	10 (7.5)	1.000
**Clinical Presentation - no. (%)**				
Fever	71 (77.2)	24 (57.1)	95 (70.9)	0.018*
Systemic symptoms (weight loss, anorexia, tiredness)	52 (56.5)	28 (66.7)	80 (59.7)	0.165
Heart murmur	57 (62)	23 (54.8)	80 (59.7)	0.431
Anemia	35 (38)	17 (40.5)	52 (38.8)	0.789
Embolization symptoms	17 (18.5)	12 (28.6)	29 (21.6)	0.188
**Number of episodes of IE- nº. (%)**				
1	84 (91.3)	38 (90.5)	122 (91)	0.816
2	6 (6.5)	1 (2.4)	7 (5.2)	
3	2 (2.2)	3 (7.1)	5 (3.7)	
**Type of IE- nº. (%)**				
Native valve	67 (72.8)	29 (69)	96 (71.6)	0.653
Prosthetic valve	21 (22.8)	13 (31)	34 (25.4)	0.316
<1yr after cardiac surgery	8 (8.7)	2 (4.8)	10 (7.5)	0.251
Device-Related Infective Endocarditis	4 (4.3)	0 (0)	4 (3)	0.309
**Valves affected - nº. (%)**				0.378
1 valve	70 (76.1)	32 (76.2)	111 (82,8)	
2 valves	12 (13)	6 (14.3)	18 (13.4)	
3 valves	1 (1)	0 (0)	1 (0.7)	
Aortic valve	53 (57.6)	24 (57.1)	77 (57.5)	0.960
Mitral valve	30 (32.6)	17 (40.5)	47 (35.1)	0.376
Right-sided valves	16 (17.4)	3 (7.1)	19 (14.2)	0.115
**Type of infection - nº. (%)**				
Community-acquired endocarditis	72 (78.2)	32 (76.2)	104 (77.6)	0.790
Healthcare-associated IE	20 (21.7)	10 (23.8)	30 (22.4)	
Continuation				
**Microbiology - nº. (%)**				
Blood culture-negative infective endocarditis	16 (17.4)	13 (31)	29 (21.6)	0.077
*Staphylococcal* species	25 (27.2)	17 (40.5)	42 (31.3)	0.124
*Staphylococcus aureus*	15 (16.3)	15 (35.4)	30 (22.4)	0.012*
*Staphylococcus epidermidis*	5 (5.4)	1 (2.4)	6 (4.5)	0.665
Other coagulase-negative *Staphylococci*	4 (4.3)	1 (2.4)	5 (3.7)	1.000
*Streptococcal* Species	34 (37)	7 (16.7)	41 (30,6)	0.018*
Viridans Group *Streptococci*	14 (15.2)	3 (7.1)	17 (12.7)	0.193
*Streptococcus gallolyticus*	12 (13)	1 (2.4)	13 (9.7)	0.063
*Streptococcus milleri*	2 (2.2)	1 (2.4)	3(2.2)	1.000
*Enterococcal* species	12 (13)	4 (9.5)	16 (11.9)	0.560
Gram-negative bacteria	2 (2.2)	3 (7.1)	5 (3.7)	0.177
Fungi	2 (2.2)	1 (2.4)	3 (2.2)	1.000
HACEK group	1 (1)	0 (0)	1 (0.7)	1.000
**Echocardiographic Findings - nº. (%)**				
Vegetation	74 (80.4)	32 (76.2)	106 (79.1)	0.776
Valve regurgitation	50 (54.3)	19 (45.2)	69 (51.5)	0.539
Valve destruction	19 (20.7)	7 (16.7)	26 (19.4)	0.722
Valve obstruction	3 (3.3)	5 (11.9)	8 (6)	0.05*
Abscess	8 (8.7)	10 (23.8)	18 (13.4)	0.009*
Pseudoaneurysm	5 (5.4)	0 (0)	5 (3.7)	0.320
Valve Aneurysm	3 (3.3)	0 (0)	3 (2.2)	0.554
Intracardiac Fistula	4 (4.3)	2 (4.8)	6 (4.5)	1.000
**Treatment - nº. (%)**				
Only medical treatment	52 (56.5)	38 (90.5)	90 (67.2)	< 0.001*
Cardiac Surgery	40 (43.5)	4 (9.5)	44 (32.8)	

* Statistically significant variable. IE: infective endocarditis; SD: standard deviation.

About half of the patients had previous arterial hypertension and valvular heart disease and 13.4% were intravenous drug users. Regarding the 13.4% of patients with Human Immunodeficiency Virus (HIV) infection, only 44% of these patients were on antiretroviral therapy at the time of the IE diagnosis; CD4 cells counts were obtained in 13 patients, with a median level of 130 ± 391 CD4 cells. About 12% of the IE cases corresponded to patients with chronic renal disease, and 31% of these were on hemodialysis.

The majority of the cases were related to native valves (71.6%), while the remaining were associated with prosthetic heart valves (25.4%) and device-related IE (3%).

Healthcare-associated infective endocarditis cases occurred in 22.4% of the patients.

About 22% of the cases had negative blood cultures. Antibiotic administration previous to blood culture collection was described in 72% of these cases. In 1 case, the IE diagnosis was made at the autopsy and blood samples were not obtained.

The most commonly isolated microorganisms were *Staphylococcus aureus* (22.4%) and Viridans Group Streptococci (12.7%).

A transthoracic echocardiography was performed in all patients, while a transesophageal study was carried out in 118 (88%) patients, with a mean time between admission and test performance of 10 ± 9.5 days (range 0-54 days). The main echocardiographic finding observed was the presence of vegetations (79.1%).

Valve regurgitation was observed in 69 cases, with 4 patients having reduced left ventricle ejection fraction (LVEF). Only 11 cases reported the LVEF and the median LVEF was 61% (IQR 18%). Systolic pulmonary artery pressure (SPAP) was reported in 15 cases, with a mean SPAP value of 41 mmHg (SD 27 mmHg).

Valve obstruction was diagnosed in 8 cases (5 cases of prosthetic IE and 3 cases in native valves) and was related to degenerated prosthesis in 4 patients, to large vegetations causing valve obstruction in 3 patients and in 1 case due to severe valvular aortic stenosis.

18F-fluorodeoxyglucose positron emission tomography/computer tomography (^18^F-FDG PET/CT) was performed in 1 patient, detecting signs of abnormal activity around the site of the prosthetic valve implantation (surgery performed more than 1 year before). None of the diagnosis was made by radiolabeled WBC single-photon emission computed tomography/Computed Tomography (SPECT/CT).

The median length of hospital stay was 41 ± 23 days (range 1-112 days). Forty-four (32.8%) patients underwent cardiac surgery. The main indication for surgery was heart failure (n = 33; 75%), followed by uncontrolled infection (n = 11; 27.3%) and prevention of embolism (n = 6; 13.6%). One patient was referred to surgery for pacemaker lead extraction. The mean time between the first day of hospitalization and surgical procedure was 26 ± 18 days, the mean time between IE diagnosis and the surgical procedure was 21 ± 16 days and the mean time between the indication for surgery and surgical procedure was 14 ± 12 days.

### Adverse outcomes during hospitalization

The in-hospital mortality rate was 31.3% (42 patients). Septic shock was the cause of death for one third of the patients (n = 14), 10 (23.8%) patients died due to heart failure, 9 (21.4%) due to embolic complications and 1 (2.4%) patient died due to cardiac tamponade. The cause of death was uncertain in 8 patients (19%).

Most of these patients (73.8%, 31 patients) were not candidates for cardiac surgery. The reasons for these patients not being candidates for cardiac surgery are described in table 2.

**Table 2:** Reasons for patients not being candidates for cardiac surgery (31 patients)

Cause	Patients (n)
Significant comorbidities:	21
Dementia and cognitive impairment with dependence in activities of daily living	5
Ischemic stroke with significant post-stroke sequelae in patients with advanced age or multiple comorbidities	5
Advanced age with substantial associated comorbidities	4
Noncompliant HIV patients with poor general clinical conditions	4
Active intravenous drug user with poor general clinical conditions	1
Malignant tumor with poor prognosis	1
Severe alcoholism with significant organ damage	1
Hemorrhagic stroke (1^st^ month after the event)	3
Active bacteremia in association with an active extracardiac infectious focus	3
Death nearly after the diagnosis (the physicians did not have the chance for surgical referral)	3
Post-autopsy diagnosis	1

Eleven patients (26.2%) were candidates for surgery but 4 died before the intervention (2 patients due to embolic events occurrence, 1 due to septic shock and 1 patient due to heart failure); 3 patients were refused for surgery by the surgical team (2 patients due to the presence of an ischemic stroke with hemorrhagic transformation and one was an HIV patient with three IE episodes that was previously submitted to 2 cardiac surgeries due to IE and with significant associated comorbidities); 4 patients died after the intervention (2 patients due to septic shock, 1 due to cardiac tamponade and in 1 the cause of death was uncertain), resulting in a surgery-related mortality rate of 9%.

The other adverse outcomes during hospitalization are described in Table 3.

**Table 3:** Adverse Outcomes during hospitalization and P value of univariate analysis of predictors of in-hospital mortality

Variable	IE Survivors (n = 92)	IE Non-Survivors (n = 42)	Total IE cases (n = 134)	p value
**In-hospital death - no. (%)**			**42 (31.3)**	
**Adverse Outcomes during Hospitalization - no. (%)**				
Heart failure	38 (41.3)	27 (64.3)	65 (48.5)	0.014*
Locally uncontrolled infection/ periannular complication	39(42.4)	16 (38.1)	55 (41)	0.639
Embolic events	30 (32.6)	21 (50)	51 (38.1)	0.054
Septic shock	8 (8.7)	19 (45.2)	27 (20.1)	< 0.001*

* Statistically significant variable. IE: infective endocarditis.

Of the 65 patients that evolved with heart failure, left ventricle systolic dysfunction was observed in 5 patients in the transthoracic echocardiography performed during hospital stay. None of the patients had previously known left ventricular systolic dysfunction.

### Predictors of in-hospital mortality

In the univariate analysis, previous heart failure, apyrexia, *Staphylococcus aureus* etiology, non-isolation of Streptococcal Species, evidence of paravalvular abscess or valve obstruction in echocardiography, incident heart failure or septic shock and absence of cardiac surgery were significantly and positively associated with in-hospital mortality (Tables 1 and 3).

In the multivariate analysis, the significant risk factors of in-hospital mortality identified in the final model were *Staphylococcus aureus* etiology, blood-culture negative endocarditis, evidence of valve obstruction in echocardiography and clinical evolution with heart failure or septic shock. Cardiac surgery was a protective factor of in-hospital mortality (Table 4).

**Table 4:** Multivariable logistic-regression model of predictors of in-hospital mortality - Final Model (including all Streptococcal Species)

Variable	Odds ratio (OR)	95% CI	p	Nagelkerke R^2^
Previous Heart Failure	3,88	0.90-16.70	0.069	0.622
Fever	0.41	0.17-1.45	0.167	
*Staphylococcus aureus*	6.47	1.07-39.09	0.042*	
*Streptococcal Species*	2.96	0.40-21.72	0.286	
Negative blood cultures	9.14	1.42-58.77	0.02*	
Valve obstruction in echocardiography	8.57	1.11-66.25	0.039*	
Abscess in echocardiography	4.14	0.89-19.21	0.07	
Heart failure	4.98	1.31-18.92	0.018*	
Septic shock	20.26	4.04-102.74	< 0.001*	
Embolic events	1.98	0.53-7.36	0.309	
Cardiac surgery	0.14	0.03-0.65	0.012*	
AUROC	< 0.001	0.88-0.97	0.926	

* Statistically significant variable. CI: confidence interval.

The model 2 that included *Streptococcus gallolyticus* organism had a numerically lower predictive performance and is described in table 5.

**Table 5:** Multivariable logistic-regression model of predictors of in-hospital mortality - Model 2 (including *Streptococcus gallolyticus* organism)

Variable	OR	95% CI	p	Nagelkerke R^2^
Previous heart failure	3.48	0.80-15.13	0.097	0.614
Fever	0.46	0.13-1.56	0.211	
*Staphylococcus aureus*	4.05	0.87-19.00	0.076	
*Streptococcus gallolyticus*	0.94	0.05-17.76	0.965	
Negative blood cultures	5.32	1.14-24.92	0.034*	
Valve obstruction in echocardiography	11.97	1.27-112.91	0.030*	
Abscess in echocardiography	3.73	0.84-16.62	0.085	
Heart failure	4.80	1.27-18.23	0.021*	
Septic shock	16.03	3.59-71.53	< 0.001*	
Embolic events	1.90	0.51-7.05	0.340	
Cardiac surgery	0.17	0.04-0.72	0.017*	
AUROC	< 0.001	0.88-0.97	0.923	

* Statistically significant variable. CI: confidence interval.

## Discussion

The factors associated to increased risk of in-hospital mortality in our cohort were: development of heart failure or septic shock, valve obstruction in echocardiography, *Staphylococcus aureus* etiology, blood-culture negative endocarditis and absence of surgical treatment.

The in-hospital mortality rate observed was 31.2%, which is slightly higher that the reported in the literature (15-30%).^3-9^

It is recognized that one of the main protective factors of mortality is cardiac surgery and it was significant in our cohort.^3,7,11-13^ Differently from other studies in which 40-50% of patients undergo cardiac surgery,^4,6,8,11,13,14^ in our center only 32.8% underwent cardiac surgery. This can be partially justified by the absence of Cardiac Surgery Department in our center, which can difficult and delay the appropriate discussion with cardiac surgeons, and subsequently it may negatively influence the in-hospital mortality rates.

The association of mortality with other factors, such as septic shock and heart failure found in our cohort is well known and expected.^3,5,8,13^

The microbiological factors that increased the risk of in-hospital mortality were the expectedly *Staphylococcus aureus*-related endocarditis^8,15^ and blood-culture negative endocarditis^14^ (possibly due to the difficulty in the diagnosis and administration of timely and directed therapy in the latter group of patients).

Valve obstruction was associated with higher mortality and in half of the patients was related to prosthesis degeneration, followed by the presence of large vegetations. In both etiologies, valve obstruction could contribute to clinical patient worsening, namely with heart failure, with congestive symptoms or low cardiac output, that could lead to multiple organ dysfunction and death.

The aortic valve was the most affected (57.5%), differently to other series in which the mitral valve was the most affected.^9^ Right-sided IE was observed in 14.2%, a value higher than the 5-10% reported.^11,16^ This could be due to the higher incidence of drug users (13.4%), compared to other series,^3,7-9,17^ which could be justified by the cultural and social characteristics of our population, and could also contribute to the higher mortality rate observed.

Prosthetic valve endocarditis occurred in 25%, in the range described in literature (10-30%).^3,7,8,13,14^

Healthcare-associated IE represents up to 30% of IE cases^8,13^ and in this study occurred in 22.4%. Agents from Staphylococcal and Streptococcal species were the most isolated microorganisms (around 30%), like expected.^3,7,8,15^ Negative-blood culture IE occurred in 21.6%, a proportion that overlaps the data found in the literature (2.1-35%).^8,14,18^

Transesophageal echocardiography (TEE) was performed in 88%. The remaining patients did not have clinical conditions to undergo a TEE or died before TEE performance. The two main echocardiographic findings were vegetations (79.1%) and valve regurgitation (51.5%).

Due to lack of ^18^F-FDG PET/CT scan and radiolabeled WBC SPECT/CT availability in our center, only 1 patient performed PET ^18^F-FDG PET/CT scan (in other center) and none performed radiolabeled leukocytes SPECT/CT.

Expectedly, heart failure was the main adverse event observed during hospitalization (48.5%).^3,8^

Complications, length of hospital stay, and mortality remain high in IE^1^ and our data highlight these facts.

This study identified the high-risk features on endocarditis patients in our cohort. The early identification of these patients might be helpful in outcome improvement by managing more closely and delivering early cardiac surgery when indicated.

These results are important not only for clinicians, once they highlighted the risk factors of death, but also to cardiac surgeons, given that they showed the good impact in prognosis of cardiac surgery.

It is important to continue with further investigations to identify other factors that could minimize the mortality levels of IE on top of the best-known management.

### Limitations

This study had a retrospective design and the information was limited to medical records. The absence of systematically collected data (such as echocardiographic measures) derived from the study design, prevented the possibility of further estimating the impact of IE in other important healthcare variables.

This study was also performed in a single center without on-site cardiac surgery and the regional variation in the diagnosis, treatment, local microbiology of IE could have influenced results and preclude the robustness of the conclusions. The sample size is unlikely to be adequately powered to assess the in-hospital mortality and risk factors. The referral bias, particularly in patients not accepted for cardiac surgery needs to be acknowledged as a limitation.

## Conclusions

According to our data, the risk factors for in-hospital mortality were the development of heart failure or septic shock, evidence of valve obstruction in echocardiography, *Staphylococcus aureus* etiology or blood-culture negative endocarditis. Invasive treatment by surgery significantly decreased the mortality risk. These results are important for all participants and emphasize the importance of having a multidisciplinary Endocarditis Team (with specialists in Internal Medicine, Cardiology, Microbiology, Infectious diseases, Cardiac Surgery) in order to address all the features associated to increased mortality.
